# Determining the Orientation of Protegrin-1 in DLPC Bilayers Using an Implicit Solvent-Membrane Model

**DOI:** 10.1371/journal.pone.0004799

**Published:** 2009-03-11

**Authors:** Abdallah Sayyed-Ahmad, Yiannis N. Kaznessis

**Affiliations:** Department of Chemical Engineering and Materials Science and the Digital Technology Center, University of Minnesota, Minneapolis, Minnesota, United States of America; Geroge Mason University, United States of America

## Abstract

Continuum models that describe the effects of solvent and biological membrane molecules on the structure and behavior of antimicrobial peptides, holds a promise to improve our understanding of the mechanisms of antimicrobial action of these peptides. In such methods, a lipid bilayer model membrane is implicitly represented by multiple layers of relatively low dielectric constant embedded in a high dielectric aqueous solvent, while an antimicrobial peptide is accounted for by a dielectric cavity with fixed partial charge at the center of each one of its atoms. In the present work, we investigate the ability of continuum approaches to predict the most probable orientation of the β-hairpin antimicrobial peptide Protegrin-1 (PG-1) in DLPC lipid bilayers by calculating the difference in the transfer free energy from an aqueous environment to a membrane-water environment for multiple orientations. The transfer free energy is computed as a sum of two terms; polar/electrostatic and non-polar. They both include energetic and entropic contributions to the free energy. We numerically solve the Poisson-Boltzmann equation to calculate the electrostatic contribution to the transfer free energy, while the non-polar contribution to the free energy is approximated using a linear solvent accessible surface area relationships. The most probable orientation of PG-1 is that with the lowest relative transfer free energy. Our simulation results indicate that PG-1 assumes an oblique orientation in DLPC lipid bilayers. The predicted most favorable orientation was with a tilt angle of 19°, which is in qualitative agreement with the experimentally observed orientations derived from solid-state NMR data.

## Introduction

Antimicrobial peptides (AMPs) are structurally diverse small proteins. They constitute an important component of the innate immune defense system in many species across the evolutionary spectrum, ranging from bacteria to mammals [1], [2]. Many of these peptides are cationic and likely to kill pathogens through nonspecifically targeting the overall anionic bacterial lipid membranes [3], [4].

Protegrin-1 (PG-1, RGGRLCYCRRRFCVCVGR-CONH2) is a cysteine-arginine rich 18 amino acid long AMP. PG-1 was originally isolated from porcine neutrophils (white blood cells) [5]. The broad strong antimicrobial, antiviral and antifungal activities of PG-1 have made it a focus of consideration as a potential starting point for designing attractive candidate antimicrobial drugs [6]–[9]. Notably, it is demonstrated that it could be administered as a topical or intradermal treatment against antibiotic resistant strains of bacteria causing skin and oral cavity infections [10], [11]. PG-1 has a β-hairpin structure that is stabilized by two disulfide bridges among its four cysteine residues. The removal of these two disulfide bridges is found to appreciably reduce the antimicrobial activity of PG-1 [12].

There is growing evidence that PG-1 acts through rapid osmotic lysis by altering the permeability of susceptible bacterial membrane. The intensity of such lysis is probably influenced by its ability to form pore-like oligomeric structures [13] which are induced through its cationic and hydrophobic content [14], [15].

The detailed mechanism of action of PG-1 and other AMPs is relatively poorly characterized due to inherent experimental limitations on obtaining atomic resolution description of the three-dimensional structure of peptides in model membrane environments. Therefore, theoretical and simulation methods [14]–[17] could provide valuable tools that potentially can be utilized to complement experimental techniques in elucidating the mechanism of action of AMPs. Of particular interest are methods based on a continuum (or implicit) description of the model membrane and the solvent molecules. Examples of these methods are the Poisson-Boltzmann [18]–[22] and Generalized-Born [23]–[25] based approaches, which have been extensively utilized to determine the orientation and the degree of insertion of AMPs and other membrane proteins in lipid bilayers. Another computationally cost-effective emerging approach is based on coarse grained modeling, whereby two or more atoms are described by a single interaction site. This cutback usually leads into a substantial reduction in the degrees of freedom of the system, thus rendering it feasible for coarse-grained models to simulate protein-membrane systems for significantly longer time scales than the ones achievable using all-atom MD simulation techniques [26]. Methods which rely on the development of residue-based or atom-based empirical potentials have been also used to predict the extent of insertion and/or the orientation of peptides in membranes [27], [28]. These methods, however, are not sensitive to variations in the local environment of the peptide and they neglect explicit electrostatic interactions. Furthermore, they depend on many adjustable parameters that are not universally optimized for all lipid bilayers. In summary, all of the aforementioned approaches may capture some features of the hydrophobicity of the lipid bilayer hydrocarbon core and the hydrophilicity of the head group region. However, they certainly neglect specific interactions, such as hydrogen bond formations and provide no relevant atomistic details regarding the solvent and membrane molecules when compared to the details that can be obtained from all-atom molecular dynamics simulations.

Resolving the positioning of AMPs in model membranes represents a crucial step in understanding the structure and behavior of these peptides. Two examples of important structural aspects of AMPs in lipid bilayers are their orientation and degree of insertion, which if elucidated, could help in determining their biological activity. Although α-helical peptides in peptide-membrane model systems have been extensively studied using approaches based on continuum description of solvent-membrane systems [18]–[20], very limited or virtually no similar investigations have been directed to study β-sheet peptides in peptide-membrane model systems. In the present study, we utilize a five slab continuum membrane-solvent model to obtain the most probable orientation of the β-hairpin AMP, PG-1, in DLPC bilayers by computing the relative transfer free energy of the peptide from solution to a given orientation in the lipid bilayer.

In the reported model, the transfer free energy is written as a sum of two terms, electrostatic and non-polar. The electrostatic contribution to the transfer free energy is calculated using the linearized Poisson-Boltzmann equation [29]–[32], while the non-polar contribution is evaluated by assuming that it is proportional to the solvent and membrane accessible surface areas of the peptide [33]. Despite the simplicity of the model used in the current study, the preferential orientation obtained using our model is found to be in good qualitative agreement with the suggested tilted transmembrane orientation observed in both widely used solid-state NMR experiments [34] and all-atom molecular dynamics simulations [15]. It is worth mentioning here that long MD simulations of peptide-membrane model systems produce results and equilibrium configurations that are dependent on the initial conditions [35]. Therefore, results obtained from an implicit model can be used to systematically provide starting configurations for full atomistic MD simulations.

## Results and Discussion

The orientation of the helix axis for α-helical membrane peptides can be adequately determined by measuring solid-state ^15^N NMR spectra. However, the case is more complex for β-sheet peptides because the N-H bonds unlike α-helical peptides are perpendicular to the principal strand axis in both transmembrane and in-plane (horizontal) membrane orientations. Both, the principal strand axis and the β-sheet plane orientations are required to resolve the orientation of β-sheet peptide in a model membrane. Yamaguchi and coworkers [34] determined the orientation of PG-1 in DLPC bilayer using solid-state ^13^C and ^15^N NMR chemical shift measurements on Val-16 ^13^CO or amid labeled peptides and the solution structure of PG-1. The reason for choosing Val-16 was because of its location in the relatively rigid part of the peptide between the between the β-turn and C-terminal.

**Figure 1:**
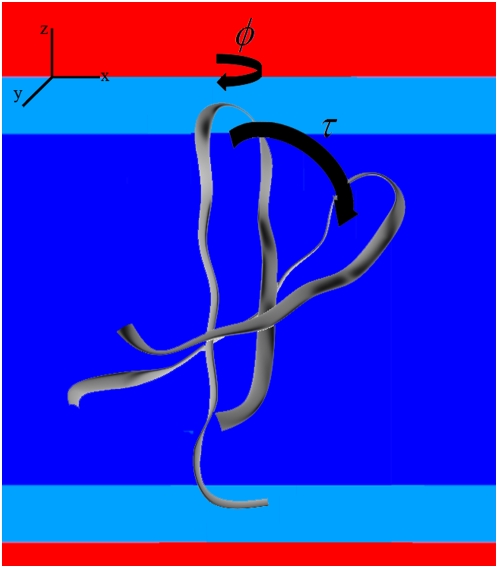
The peptide orientation is determined by two angles. The first angle is the tilt angle (τ) which is defined as the angle between the principal axis of the peptide backbone and the normal to the plane of the lipid bilayer, while the second angle is the rotation angle (φ) which is defined as the angle between the normal to the β-sheet plane and the vector normal to the lipid bilayer.

Since the orientation of the peptide relative to the bilayer normal is dependent on the definition of the β-hairpin axis and the β-sheet plane, we determine the peptide orientation by two orthogonal angles as demonstrated in Figure 1. The first angle is the tilt angle (τ) which is defined as the angle between the principal axis of the peptide backbone and the normal to the plane of the lipid bilayer, while the second angle is the azimuthal rotation angle (φ) which is defined as the angle between the normal to the β-sheet plane and the vector normal to the lipid bilayer. The C and N terminal and nearby residues of PG-1 sample multiple conformations in the solution NMR structures[36] and the DLPC bilayer MD simulation [15]. Therefore, the β-sheet plane was determined by a least square fitting through all C_α_ atoms of the relatively rigid core of the peptide (residues 4 to 16).

We should note that it may be difficult to accurately compute absolute transfer free energies of an AMP from the bulk aqueous phase to a model membrane. The model used in the current study does not explicitly consider many effects that could contribute to the net transfer free energy such as lipid molecule perturbation effects, peptide conformational changes, peptide immobilization and the resultant entropic loss. However, calculations of relative transfer free energy of different orientations as compared to a reference orientation are expected to be more accurate since it is presumed that most systematic errors in the model and calculations will cancel out. Based on previous MD-simulations and experimental evidence, PG-1 is assumed to penetrate the DLPC bilayer and reside in the membrane [15], [34]. Therefore, regardless of the insertion process, the orientation of an AMP depends mainly on the interaction of the peptide with the lipid molecules. We calculate, 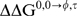, the transfer free energy for a given peptide conformational orientation (φ, τ) relative to a reference orientation (0, 0) to determine the most probable orientation as follows
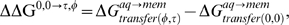
(1)where 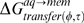 is the transfer free energy of the peptide from the aqueous state to a (φ, τ) orientation in the membrane.

**Figure 2:**
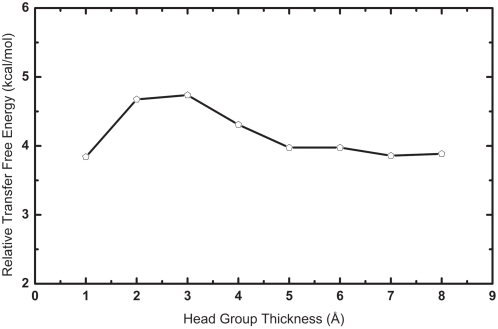
The transfer free energy of PG-1 conformation with a tilt angle of 15° relative to the conformation with a tilt angle of 0° as function of the head group region thickness.

The positively charged arginine residues located at the β-turn and termini regions, as well as the significant hydrophobic patch at the center of PG-1 give it an amphipathic nature that may contribute to its biological properties and allow it to interact with both the non-polar hydrocarbon core of the membrane and the polar head group region. The transfer free energy is therefore dependent on the dielectric property and the thickness of the lipid bilayer regions. The membrane environment as we mentioned earlier is described by multiple slabs of dielectric environment. The thickness of the hydrocarbon core slab depends on the type of the bilayer modeled and the other physicochemical conditions. In this study for a DLPC bilayer, we assigned its hydrocarbon core region with a 30 Å thick low dielectric region. Figure 2 shows the relative transfer free energy between a tilt of 15° and 0° as a function of the head group region thickness in Å. The difference in the transfer free energy stabilizes beyond the thickness of 5 Å, which is the value used in this study to determine the most favorable orientation of PG-1 in the DLPC bilayer.

**Figure 3:**
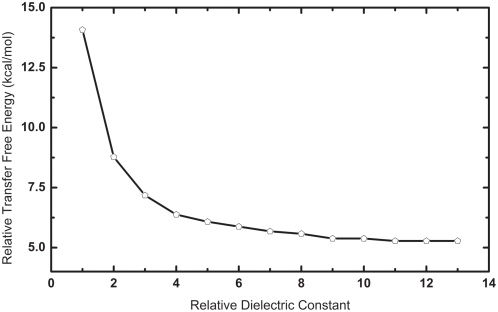
The transfer free energy of PG-1 conformation with a tilt angle of 15° relative to the conformation with a tilt angle of 0° as function of the relative dielectric constant of the head group region.

There have been multiple values of the dielectric constant reported in the literature for proteins and lipid bilayers which makes it difficult to define unique values for the peptide and both the hydrocarbon core and head group regions. In the present study, we assign the interior of the peptide with a dielectric constant of 1.0. The non-polar hydrocarbon core were assigned a low relative dielectric constant of 2.0 [37]–[39], whereas the solvent region was assigned a relative dielectric constant of 80. The value for the relative dielectric constant for the head group region is not well characterized, thus it is questionable whether the head group region can be assigned with a uniform relative dielectric constant [33], or even by multiple layers with different relative dielectric constants as adopted by Tanizaki and Feig [40]. Figure 3 indicates the transfer free energy of PG-1 conformation with a tilt angle of 15° relative to the conformation with a tilt angle of 0° as function of the relative dielectric constant of the head group region. It clearly shows that the effect of change in the relative dielectric constant of the head group region on the relative transfer free energy diminishes beyond the value of 10. Although not conclusive because of the need to have a more comprehensive sampling over all angles, in the current study, we assign the head group region with an intermediate relative dielectric constant of 10.0 similar to the value adopted by Sengupta and coworkers [18].

**Figure 4:**
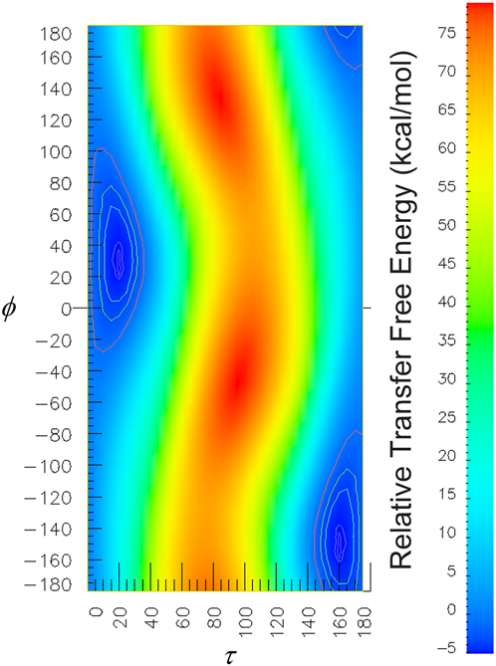
Color coded map of the transfer free energy of all PG-1 conformations with a tilt angle (τ) and the rotation angle (φ) relative to the reference conformation with tilt and rotation angles of 0°.

**Figure 5:**
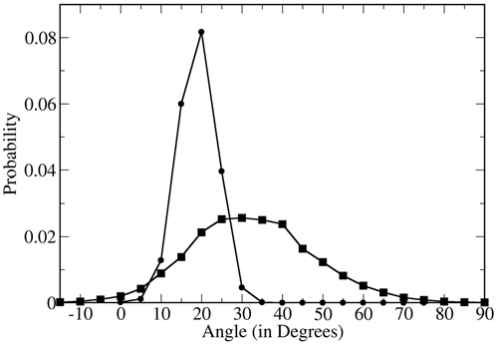
The marginal probability densities of the tilt angle and azimuthal rotation angle as indicated in solid circles and solid squares, respectively.

We carried out a comprehensive search over the two angles (φ, τ) with a 5° resolution to determine the most probable orientation of PG-1 in DLPC bilayer. Figure 4 demonstrates a color coded map of the transfer free energy of all PG-1 conformations with a tilt angle (τ) and the rotation angle (φ) relative to the reference conformation with tilt and rotation angles of 0°. It indicates that a horizontal orientation of the PG-1 in DLPC bilayer is highly unfavorable because of the higher transfer free energy for such orientation relative to the reference orientation. Figure 5 shows the marginal probability densities of both the tilt and azimuthal rotation angles. It also indicates that the most probable tilt angle for PG-1 in DLPC bilayer is around 20°, while the most probable rotation angle between the normal to the β-sheet plane and the vector normal to the lipid bilayer is approximately 30°. The transfer free energy for this orientation is 5.18 kcal/mol lower than the reference vertical orientation with 0° tilt and rotation angles.

The expected value of the tilt angle and the azimuthal rotation angle are computed using a Boltzmann weighted average as follows
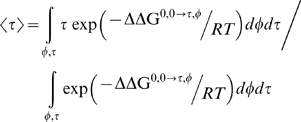
(2)and
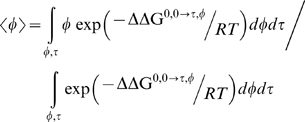
(3) The expected values of the two angles are found to be approximately 19° and 32.5°, respectively.

The expected value of the tilt and the azimuthal rotation angles are indeed in a qualitative agreement with the experimental NMR derived orientation found by Yamaguchi and coworkers, who reported a tilt angle of approximately 50±5° and a rotation angle of 48±5°. One reason for this quantitative discrepancy is due to the fact that the transfer of PG-1 from the aqueous medium to the membrane environment could result in conformational changes. The most probable orientation obtained using our method and even from NMR data [34] depend on the backbone and side-chain conformations. The conformation of the β-hairpin used in the calculations to obtain the experimental values of the tilt and azimuthal rotation angles was based on the most regular NMR solution structure. Using other solution structures lead to a larger uncertainty in the orientation of the peptide which exceeds any experimental errors in the solid-state NMR data [34].

Regular structures, which are most likely to be adopted in the lipid bilayer than in the aqueous solution as suggested by MD simulations and experimental data show that conformational changes of the backbone of PG1 when immersed in a lipid bilayer are relatively small due to its structural stabilization by the two disulfide bridges in the rigid part of the peptide (residues 4–16) [15], [34]. Most importantly, the model developed here was able to capture that PG-1 adopts a more favorable oblique orientation in DLPC bilayer than a horizontal one, which indicates the utility of using the approach to study membrane-protein systems.

An important aspect of the mechanism of action of antimicrobial peptides is their ability to form pores that leads to osmotic lysis of cells. The model developed here has the potential to be used in studying the formation free energy of oligomeric structures of PG-1 and other AMPs in model membranes, and thereby provides a method that could be used to screen different mutants according to their antimicrobial activities. Furthermore, results obtained from an implicit model can be used to systematically provide starting configurations for full atomistic MD simulations. Developing new and validating existing models that address the challenge of predicting the structure and orientation of AMPs and other membrane proteins in model membrane systems is, therefore, of fundamental importance to the fields of molecular modeling, computational biology and biotechnology.

## Materials and Methods

### PG-1 Structure

The initial structure of PG-1 was obtained from the RCSB Protein Data Bank (pdb ids: 1pg1). These experimental structures were originally acquired from multiple samples analyzed using two-dimensional homonuclear magnetic resonance spectroscopy at a peptide molar concentration of 2.0–4.0 mM dissolved in water or 10 mM sodium phosphate solution [36]. The ultimate structure investigated in the current study was extracted from the last snapshot of our previous 24 ns explicit-solvent molecular dynamics simulation [15]. A constrained minimization using few steps of steepest descent method was carried out to insure the removal of any close bad contacts among the peptide atoms. The ionizable groups of PG-1 were protonated or deprotonated assuming the pH for the unbound peptide and peptide in the membrane model to be equal to 7.0. The NH_2_ terminus was protonated, while the C-terminus was blocked by neutralizing its negative charge through amidation. Thus, the six positively charged arginine residues and the positively protonated N-terminus give PG-1 peptide in solution a net charge of +7 e. The protonation states of the membrane-embedded residues are assumed to remain unchanged.

### Transfer Free Energy Calculations

It has been previously shown that the transfer free energy of a peptide from the aqueous solution state to the membrane embedded state, 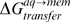, can be approximated as a sum of two main contributions as follows [19], [33]

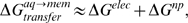
(4) where 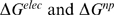 are the change in the electrostatic and non-polar contributions to the free energy upon the transfer of the peptide from bulk water to membrane environment. Other contributions to the transfer free energy that account for processes such as peptide immobilization, lipid molecule perturbation and peptide conformational changes are not included in this study. The rationale behind these approximations is discussed in a brief detail in the results and discussion section.

Biological membranes are very complex fluid systems that are usually composed of a mixture of lipids and some membrane proteins. The structural properties of these membranes are commonly derived from the bilayer arrangement of the constituent lipids whereby the polar head groups of the lipid molecules are exposed to the aqueous phase, while the hydrophobic lipid hydrocarbon tails bring about a central non-polar interior. Here, we adopt an implicit membrane model structure similar to the one used by Sengupta and coworkers [18] to capture these essential aspects of model membrane bilayers. The biological membrane in this model is represented by a simplified description using three structureless homogeneous slabs of dielectric continuum with a low relative dielectric constant assigned to the hydrocarbon region due to its low molecular polarizability [37], whereas the polar head group region is assigned with a higher relative dielectric constant [41] that depends on many factors such as the composition of the head group and its packing density.

**Figure 6:**
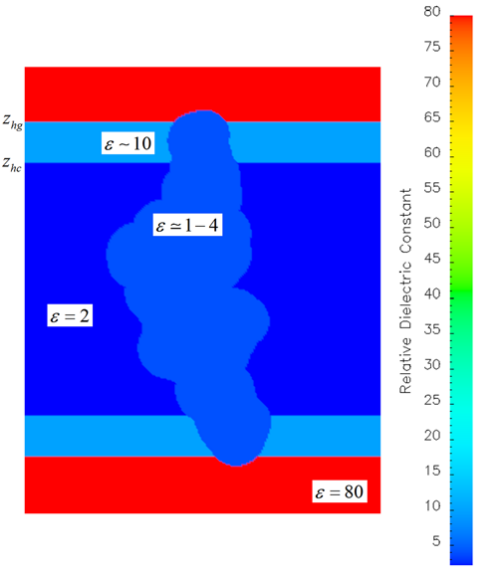
The 5 slab membrane model used in the current study with the relative dielectric constant along the direction normal to the model membrane plane.

The numerical solution of the linear Poisson-Boltzmann equation provides an approximation to the electrostatic contribution to the change in the free energy upon transferring the peptide from the aqueous solution to the membrane environment. In the Poisson-Boltzmann approach, the atoms of the AMP can be represented by a dielectric cavity with explicit fixed charges on the center of its atoms, while the solvent and the model membrane can be described by homogeneous continuum dielectric slabs as schematically indicated in Figure 6. The implementation details of the calculations are as follows.

The electrostatic contributions to the solvation and desolvation free energies were approximately obtained by solving the linearized Poisson-Boltzmann equation on a 251^3^ grid and using the PBEQ module [31] as implemented in the CHARMM program version c30b [42]. The atomic coordinates and radii of the atoms of the peptide were used to define the dielectric cavity region as the volume inaccessible to contact by a sphere of radius 1.4 Å rolling over the peptide molecular surface. The radii were taken from the optimized atomic radii set for Poisson-Boltzmann calculations, obtained by Nina and coworkers [43]. The ion exclusion radius was set to 1.5 Å and the ionic strength was set to 0.15 M in the aqueous environment to account for physiological salinity conditions. The electrostatic potential was computed using a hexahedral mesh with a grid space of 0.25 Å and the Debye-Hückel approximation to estimate the boundary conditions in the direction normal to the model membrane surface and periodic boundary conditions the membrane plane. The calculations were carried out at temperature of 298 K and no membrane potential was applied in the implicit membrane model. The bilayer was centered at the plane z = 0. Similarly, the center of mass of the backbone atoms of the peptide also was aligned to go through the same plane.

The non-polar contribution to the transfer free energy is essentially important in determining the most probable orientation in heterogeneous environment like lipid bilayers. It includes contributions that account for the cost of cavity formation and the van der Waals interactions with the solvent and membrane molecules. The non-polar term is assumed to be proportional to the solvent-accessible surface area. For a peptide with 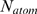 atoms, it is approximated using the linear relationship
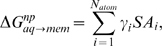
(5) where 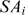 is the solvent accessible surface area, which obtained using a probe radius of 1.4 Å ; 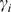 is an empirical surface tension parameter assigned for the *i*-th atom with a continuous transition from the hydrocarbon core to the aqueous phase through the head group region as follows
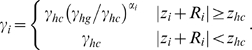
(6)

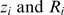
 are the z-coordinate and the van der Waals radius of the *i*-th atom, respectively, while 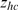 is half the hydrocarbon core region thickness. The parameter associated with the hydrocarbon core region, 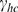, is set to equal −0.0278 (cal mol^−1^Å^−2^). This parameter has been estimated from the partitioning of alkanes between liquid alkane and water [44]. There are other phenomenological forms reported in the literature to compute the nonpolar contribution to the transfer free energy and capture the gradual transition from the bilayer to the bulk solvent, however there is no consensus on the shape of the transition function or a rigorous derivation to obtain it [25], [45]. The extent of non-polar contributions associated with the insertion of a peptide in the head group region is expected to be smaller than the one associated with similar insertion in the hydrocarbon core due to the polar nature of the head group region. The parameter associated with the polar head group region, 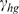, is set to −0.012 (cal mol^−1^Å^−2^) [33]. The exponent parameter, 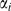, is calculated using the following formula
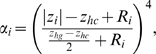
(7)where 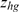 is half thickness of the membrane. Note that 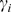 is equal to 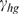 for atoms with their center coinciding with the equator plane of the polar head group region and it gradually vanishes as the center of an atom moves away from that equator.
